# Comparative prostate MRI before and after chronic granulomatous prostatitis following intravesical bacillus Calmette–Guérin therapy

**DOI:** 10.2144/fsoa-2020-0065

**Published:** 2020-10-20

**Authors:** Julien Sarkis, Georges Nawfal, Elias El-Haddad, Georges Abi Tayeh, Nathalie Mahfoud, Pierre Sarkis

**Affiliations:** 1 Department of Urology, Hôtel-Dieu de France Hospital, Beirut, Lebanon; 2 Department of Radiology, Saint Joseph Hospital, Beirut, Lebanon; 3 Department of Radiology, Hôtel-Dieu de France Hospital, Beirut, Lebanon; 4 Department of Pathology, Saint Joseph Hospital, Beirut, Lebanon; 5 Department of Urology, Saint Joseph Hospital, Beirut, Lebanon

**Keywords:** BCG therapy, granulomatous prostatitis, prostate cancer, prostate MRI, prostate-specific antigen, PSA

## Abstract

**Background:**

Granulomatous prostatitis (GnP) is an interesting complication of bacillus Calmette–Guérin (BCG) therapy as it mimics prostate cancer on clinical, biochemical and imaging examinations. In the era of multiparametric prostate MRI (mpMRI), differentiation of GnP from prostate cancer on imaging is essential.

**Case presentation:**

We report a case of post-BCG GnP in a patient with nonmuscle invasive bladder cancer, presenting with a prostate-specific antigen level of 21.6 ng/ml and prostate imaging reporting and data system (PI-RADS) 5 peripheral lesions. A mpMRI performed 6 months before showed a score 2 of PI-RADS.

**Conclusion:**

The comparison of mpMRI images before and after BCG administration gives urologists, oncologists and radiologists a precise idea of the mpMRI changes that occur following BCG administration to eventually prevent unnecessary biopsies in future patients.

## Lay abstract

Granulomatous prostatitis is a bacillus Calmette–Guérin therapy complication that often mimics prostate cancer. This article gives radiologists, urologists and oncologists a clear multiparametric prostate MRI comparison of the changes that bacillus Calmette–Guérin prostatitis causes on the prostate gland.

Bacillus Calmette–Guérin (BCG) is recommended as intravesical immunotherapy in intermediate- and high-risk nonmuscle invasive bladder cancer [1]. Granulomatous prostatitis (GnP) is an interesting complication of BCG therapy, accounting for approximatively 1.3% of patients [2]. It mimics prostate cancer on clinical, biochemical and imaging examinations [3]. Despite the recent advances in prostate imaging, the MRI appearance of GnP after BCG therapy is not well described due to its relative rarity [4].

We herein present a case of GnP diagnosed 2 months after completion of a 6-week course of BCG, on the basis of high prostate-specific antigen (PSA) and score 5 of prostate imaging reporting and data system (PI-RADS) on multiparametric prostate MRI (mpMRI). Of note, a previous mpMRI performed 6 months before showed a score 2 of PI-RADS.

## Case presentation

**Figure 1.**
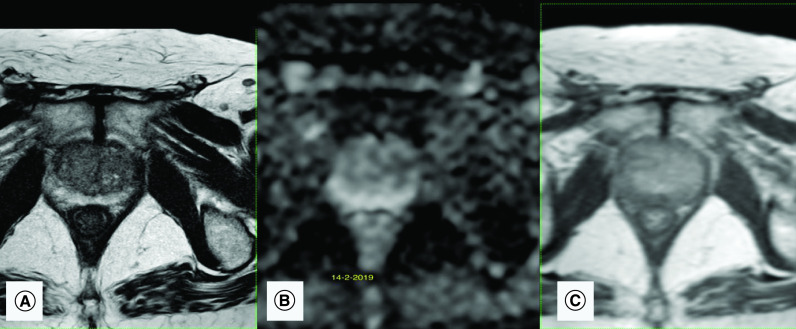
Multiparametric prostatic MRI before intravesical bacillus Calmette–Guérin therapy. **(A)** Axial T2-weighted image shows peripheral symmetrical hypointense linear lesions corresponding to sequelae of chronic prostatitis. **(B)** Apparent diffusion coefficient map of the diffusion-weighted image shows no corresponding diffusion restriction in peripheral zones. **(C)** Dynamic contrast enhancement shows only mild enhancement mainly in the left peripheral zone. These findings suggest sequelae of chronic prostatitis and a PI-RADS score of 2.

A 72-year-old male patient presented for elevated PSA of 6.5 ng/ml (normal PSA <4.0 ng/ml) with a normal digital rectal exam. A mpMRI performed showed benign (PI-RADS 2) lesions at the peripheral zone (Figure 1). Consequently, prostate biopsies were deemed unnecessary and a PSA follow-up decided after 6 months.

A month later he developed isolated macroscopic hematuria with work-up revealing an intermediate-risk nonmuscle invasive bladder cancer (multifocal low grade bladder tumor pTa LG) managed with a complete transurethral resection of the bladder and subsequent 6-week course of intravesical BCG therapy.

**Figure 2.**
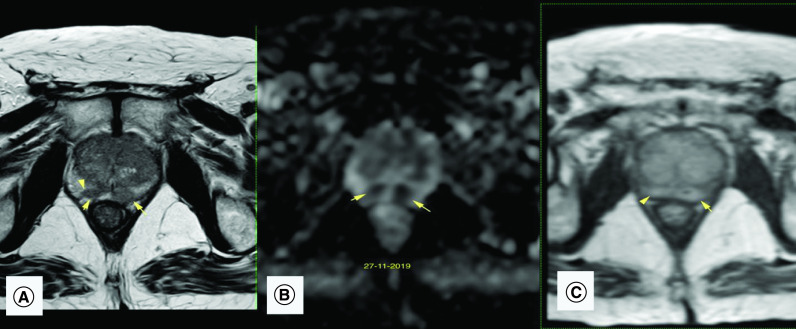
Multiparametric prostatic MRI after intravesical bacillus Calmette–Guérin therapy. **(A)** Axial T2-weighted image: a focal and ill-defined hypointense lesion in the right and left PZ (arrow) exhibits a bulging contour (arrowhead), suspicious of prostate cancer. **(B)** Apparent diffusion coefficient map: the PZ lesions (arrows) exhibit very low signal intensity in the apparent diffusion coefficient map. This is compatible with significant diffusion restriction in those areas. **(C)** Dynamic contrast enhancement shows moderate to significant early and diffuse enhancement mainly in the PZ. These findings are compatible with a PI-RADS score of 5. PZ: Peripheral zone.

**Figure 3.**
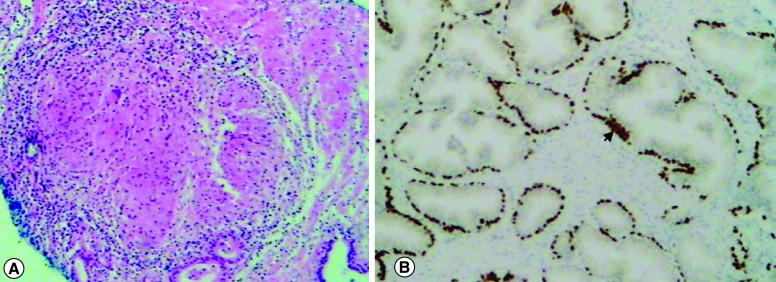
Histopathological findings of in-bore prostate biopsy. **(A)** Hematoxylin and eosin staining showing a non-necrotizing granuloma composed of epithelioid histiocytes and giant cells. **(B)** Immunochemistry highlighting normal prostatic glands with positive p63 basal cells (arrowhead), not in favor of prostate cancer.

During his follow-up 2 months after his last BCG instillation, PSA level rose to 21.6 ng/ml, with a repeated dosage of 22 ng/ml after 2 weeks. The patient was asymptomatic. Urine culture was negative and rectal examination was not suspicious. A new mpMRI showed multiple PI-RADS 5 lesions in the right and left peripheral zone (Figure 2). Despite the clinical suspicion of granulomatous prostatitis, patient anxiety as well as high PSA levels pushed us toward performing prostate biopsies. MRI guided in-bore prostate biopsies of the lesions were realized, showing an inflammatory process with noncaseating granulomatous reaction with no sign of malignancy (Figure 3), all in favor of granulomatous prostatitis secondary to intravesical BCG therapy. No antituberculous treatment was started. Patient was followed up with a PSA of 14.4 ng/ml after 3 months.

## Discussion

Intravesical instillation of BCG is recommended following transurethral resection of the bladder in the setting of intermediate- and high-risk nonmuscle invasive bladder cancer, reducing cancer progression and recurrence [1]. Although rare, adverse effects of BCG therapy can range from local side effects like BCG cystitis, to more serious systemic BCG infections [5]. In addition, over 40% of patients present a significant increase in their PSA level [6,7] with prostatic granulomas often mimicking prostatic neoplasm on clinical [8] and imaging examinations (accounting to over 6% of PI-RADS-5 lesions on mpMRI [9]).

Few reports describe the MRI features of GnP, which are nonspecific. In a retrospective case series of ten patients, Suzuki *et al.* concluded that GnP generally appears hypointense relative to the surrounding peripheral zone on T2-weighted images and hyperintense on diffusion-weighted images [10], without mentioning the corresponding PI-RADS score. This appearance is variable depending on the amount of free radicals produced by the inflammatory process [4,10]. With dynamic contrast enhancement, prostatic granulomas do not exhibit the rapid wash-in and wash-out seen in prostate cancer. However, indeterminate abnormalities would almost certainly require biopsy regardless of how well the imaging features are analyzed, especially in those with elevated PSA [11].

What is interesting in our patient is that a previous mpMRI had been done 4 months ago before BCG therapy showing a basically normal to mild prostatic abnormalities (score 2), with prominent nodular markedly hypointense diffuse lesions appearing on the latter MRI done after BCG therapy (score 5). Comparing both mpMRI can give urologists and radiologists a more precise idea of the imaging changes following BCG administration to eventually prevent unnecessary biopsies in future patients.

In addition, in a prospective study by Leibovici *et al.*, PSA levels rose to a maximal average of 6.97 ng/ml during BCG treatment [4]. Our patient presented a PSA elevation to 22 ng/ml, higher than the values reported in the literature pushing us toward performing prostate biopsies.

In this case, we only performed a targeted prostate biopsy. This could be debatable as extended plus targeted biopsy are strongly recommended at first presentation. However, our patient had a focal PI-RADS-5 lesion on mpMRI and the diagnosis of granulomatous prostatitis was highly probable considering the chronology of events. In their study, Grey *et al.* found that PI-RADS-5 lesions had specificity of 96.5% (95% confidence interval [CI] 91.7–98.7) for prostate cancer [12]. Therefore, to avoid the number of biopsy scores and to lower the risk of prostate biopsy complications, only targeted biopsies were performed.

No clear guidelines exist concerning the correct treatment of granulomatous prostatitis. Since our patient had no urinary or systemic symptoms and because of the absence of caseous necrosis on histology, he was managed conservatively with PSA follow-up and without antituberculosis treatment [13].

## Conclusion & future perspective

GnP is a known complication of intravesical BCG. When it is associated with a high PSA and a suspicious clinical exam, it can easily be confounded with prostate cancer. The current report has the advantage of exposing the exact mpMRI changes following BCG, as the patient had an MRI before and after BCG administration. Additional comparative MRI analysis are of course needed, to reduce the number of unnecessary biopsies in GnP patients.

**Executive summary**

- Granulomatous prostatitis (GnP) is an interesting complication of BCG therapy that mimics prostate cancer on clinical, biochemical and imaging examinations.
- We present a case of GnP diagnosed 2 months after completion of a 6-week course of BCG, on the basis of high prostate-specific antigen and score 5 of PI-RADS on multiparametric prostate MRI (mpMRI). Of note, a previous mpMRI performed 6 months before showed a score 2 of PI-RADS.
- Few reports describe the MRI characteristics of GnP, which are nonspecific. Our case describes the exact features of GnP on mpMRI.
- No clear guidelines exist concerning the correct treatment of GnP. Our patient had an asymptomatic GnP. The patient was managed conservatively without antituberculosis therapy, with a decrease of his prostate-specific antigen after 6 months.
